# Syndrome de Sweet: étude clinique et natomopathologique sur 5 ans

**DOI:** 10.11604/pamj.2015.20.362.4274

**Published:** 2015-04-14

**Authors:** Hanae Bouzidi, Salim Gallouj, Nissrine Amraoui, Fatima Zahra Mernissi, Taoufiq Harmouch

**Affiliations:** 1 Service de Dermatologie-Vénérologie, CHU Hassan II, Fès, Maroc; 2 Service d'Anatomopathologie, CHU Hassan II, Fès, Maroc

**Keywords:** Syndrome de Sweet (SS), dermatose aiguë fébrile, lésions de vascularite, Sweet Syndrome:, acute febrile dermatosis, vasculitis

## Abstract

Le syndrome de Sweet ou dermatose aiguë fébrile neutrophilique est une maladie inflammatoire rare à expression cutanée prédominante, appartenant au groupe des dermatoses neutrophiliques. Il est caractérisé par le polymorphisme de son expression clinique et la diversité des maladies qui peuvent lui être associées. Le but de ce travail était d’étudier les particularités cliniques, anatomopathologiques, thérapeutiques et évolutives du syndrome de Sweet. Nous rapportons une étude rétrospective et descriptive de 25 cas de syndrome de Sweet observés dans les services de dermatologie et d'anatomie pathologique du centre hospitalier universitaire de Fès sur une période de 5 ans. Notre série était constituée de 5 hommes et de 20 femmes avec un sex-ratio (hommes/femmes) de 0,25. L’âge moyen des patients était de 47 ans avec des extrêmes allant de 11 à 75 ans. Des maladies associées étaient retrouvées chez 17 patients: hémopathies (deux cas), tumeur solide (1cas), maladie chronique de l'intestin (1cas), tuberculose (1 cas), diabète (trois cas) et infections (9 cas). Deux patientes étaient enceintes au moment du diagnostic. Les manifestations cutanées étaient polymorphes avec atteintes muqueuses dans deux cas. Les lésions siégeaient le plus souvent au niveau acral. Histologiquement, le derme était le siège d'un infiltrat dense et diffus riche en polynucléaires neutrophiles dans tous les cas. Une inflammation de la paroi des vaisseaux étaient observées dans trois cas. Le syndrome de Sweet peut être révélateur ou précéder des maladies associées, ce qui impose une surveillance rigoureuse et prolongée.

## Introduction

Le syndrome de Sweet (SS), ou dermatose aiguë fébrile neutrophilique, a été décrit pour la première fois par Robert Douglas Sweet en 1964 [1]. Il appartient au groupe des dermatoses neutrophiliques. Il peut être considéré comme la plus typique des entités de ce groupe [2–4]. Il est caractérisé par le polymorphisme de son expression clinique et la diversité des maladies qui peuvent lui être associées. Il se manifeste classiquement par une fièvre, une éruption de plaques ou de nodules érythémateux cutanés, une hyperleucocytose à polynucléaires neutrophiles (PNN) et un infiltrat dermique massif constitué essentiellement de PNN. Le traitement repose sur des traitements anti-inflammatoires qui permettent une résolution rapide des symptômes et des lésions cutanéomuqueuses [2]. Nous étudierons les caractéristiques cliniques, anatomopathologiques, thérapeutiques et évolutives de cette affection à partir d'une étude rétrospective et descriptive d'une série de 25 cas de SS.

## Méthodes

Les dossiers de 25 cas de SS diagnostiqués dans notre institution du 1^er^ janvier 2009 au 1^er^janvier 2014 ont été revus. Une fiche analytique complète a été établie pour chaque patient. Tous les patients ont eu un examen clinique complet, une numération de la formule sanguine, une vitesse de sédimentation, un dosage de la protéine C-réactive (CRP), et une biopsie cutanée. Le diagnostic positif de SS était établi sur les critères cliniques, biologiques et histologiques (Tableau 1). Le diagnostic a été retenu devant la présence de deux critères majeurs et deux critères mineurs. Le syndrome d'immunodéficience acquise a été recherché chez tous nos patients par une sérologie HIV, les hémopathies par une EEP, Les autres maladies associées ont été recherchées par des explorations biologiques et radiologiques en fonction des signes d'appel. Nous avons procédé au cours de cette étude à une analyse détaillée de notre série en fonction de l’âge, du sexe, des antécédents, de la présentation clinique, des données biologiques, de l'aspect anatomopathologique, du traitement et des différentes modalités évolutives.

**Tableau 1:** Critères diagnostiques du syndrome de Sweet [5]

Critères majeurs	Critères mineurs
Éruption brutale de plaques ou de nodules érythémateux, sensibles ou douloureux	Fièvre > 38°C
	L'association à une maladie inflammatoire, un syndrome myéloprolifératif, une tumeur maligne solide, une vaccination, une grossesse ou précédé par une infection gastro-intestinale ou respiratoire
Infiltrat dermique dense à polynucléaires neutrophiles	Très bonne réponse à la corticothérapie systémique
	Anomalies biologiques (trois critères sur quatre): VS > 20 mm/h; Elévation de la CRP; hyperleucocytose > 8000/mm^3^ ; neutrophiles > 70%

## Résultats

**Figure 1:**
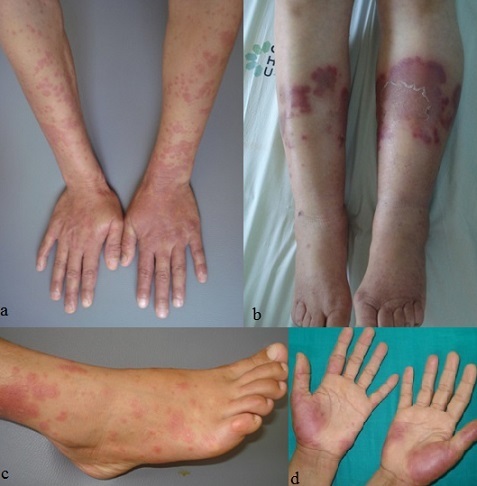
(a, b, c, d) présence de papules et plaques érythémateuses œdémateuses au niveau acral chez des patients présentant un syndrome de sweet

**Figure 2:**
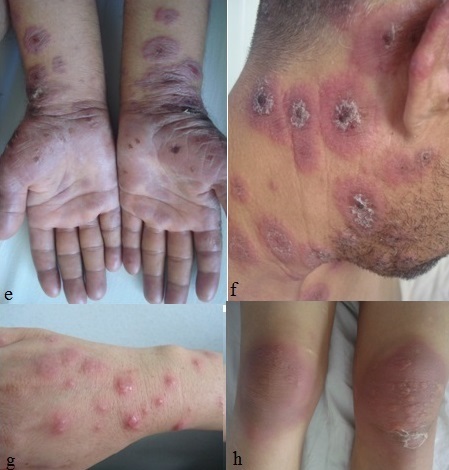
(e, f) lésions en cocarde crouteuses; (g, h) lésions vésiculeuses et bulleuses dans le cadre de la maladie de sweet

Entre le mois de janvier 2009 et le mois de janvier 2014, nous avons colligé 45 cas de dermatoses neutrophiliques qui se répartissaient comme suit: 25 cas de SS (55,5%), 15 cas de pyoderma gangrenosum (33,3%), 3 cas de pustulose sous-cornée (6,6%), 2 cas d'erythema elevatum diutinum (4,4%). Notre série de SS était constituée de 5 hommes et 20 femmes avec un sex-ratio (homme/femme) de 0,25. L’âge moyen des patients était de 47 ans avec des extrêmes allant de 11 à 75 ans et un pic entre la quatrième et la cinquième décennie. Des maladies associées étaient retrouvées chez 17 patients. Il s'agissait d'infections dans 9 cas (angines, érysipèle, cellulite frontale et maxillaire, sinusite et dacryocystite), une hémopathie dans 2 cas (leucémie lymphoïde chronique et leucémie myéloïde aigue), une tumeur solide dans un cas (adénocarcinome mammaire), une maladie cœliaque dans un cas, une tuberculose dans un cas et un diabète dans trois cas. Deux cas étaient associés à une prise médicamenteuse (cycline et quinolone). Par ailleurs, deux femmes étaient enceintes, à 16 et 28 semaines d'aménorrhée. La symptomatologie était d'installation brutale dans tous les cas. Le délai entre le début des premiers symptômes et la première consultation variait entre un jour et trois mois. À la phase prodromique, une symptomatologie fonctionnelle était rapportée dans 21 cas (84%). Il s'agissait d'une fièvre (19 cas), d'un syndrome pseudogrippal (sept cas), d'arthralgies (10 cas), d'une conjonctivite (5 cas), ou d'une toux sèche (un cas). À la phase d’état, une éruption cutanée était présente chez tous les patients. Elle consistait en des plaques papuleuses érythémateuses ou violines infiltrées bien limitées, de taille variable, dans 19 cas (Figure 1). Des aspects atypiques étaient présents dans 18 cas: des lésions en cocarde ou en pseudo-cocarde (7cas), des bulles (4 cas), des pustules (4 cas) ou des vésicules (deux cas) (Figure 2). Ces lésions étaient retrouvées sur les membres supérieurs dans 15 cas, sur les membres inférieurs dans 11 cas, dans la région de la tête et du cou dans 19 cas et du tronc dans deux cas. 2 atteintes muqueuses étaient notées dans notre série. La répartition des manifestations extra-cutanées chez les patients était par ordre de fréquence décroissant: osteoraticulaire dans 14% des cas, oculaire 10%, rénal dans 7% des cas, neurologique, musculaire et pulmonaire dans 3% des cas chacun.

**Figure 3:**
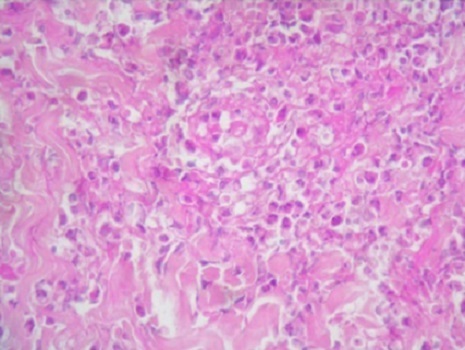
Lésions de vascularite leucocytoclasique de la paroi des vaisseaux (HE x 40)

La numération formule sanguine montrait une hyperleucocytose dans 16 cas (64%). Cette hyperleucocytose était à prédominance de polynucléaires (PNN) (supérieure à 70%). La vitesse de sédimentation à la première heure était augmentée dans tous les cas avec une moyenne de 85,1 mm/1 h et des extrêmes allant de 10 à 140 mm/1 h. La valeur moyenne de la CRP était de 57,5 mg/L (6-194). Sur le plan anatomopathologique, l’épiderme était normal dans 13 cas (52%). Des modifications épidermiques étaient retrouvées dans 12 cas (48%). Elles consistaient en une hyperkératose orthokératosique (20%), une acanthose (12%), une pustule spongiforme (16%) ou une spongiose (4%). Le derme réticulaire était le siège d'une inflammation dense dans tous les cas. L'infiltrat était constitué de PNN dans tous les cas, mais associait également des polynucléaires éosinophiles dans 5 cas (20%), des éléments lymphoplasmocytaires dans 10 cas (40%) et des histiocytes dans 3 cas (12%). Des débris nucléaires de PNN étaient notés dans 5 cas (20%). Au niveau du derme papillaire, un œdème était retrouvé dans 12 cas (48%) et une vasodilatation dans 11 cas (44%). Une vascularite leucocytoclasique était présente dans 3 cas (12%) (Figure 3). L'hypoderme, prélevé dans la majorité des cas, était d'aspect normal (Tableau 2).

**Tableau 2:** Tableau récapitulatif des lésions histologiques observées chez 25 patients avec syndrome de sweet

Lésions histologiques	Nombres de cas	Pourcentage
**Epiderme**		
Normal	13	52
Modification épidermiques	12	48
**Derme**		
Polynucléaires neutrophiles (PNN)	25	100
Polynucléaires éosinophiles	5	20
Eléments lymphoplasmocytaires	10	40
Histiocytes	3	12
Débris de PNN	5	20
Œdème	12	48
Congestion vasculaire	11	44
Vascularite leucocytoclasique	3	12

L'aspect histologique était typique d'un SS dans la grande majorité des cas. Les problèmes de diagnostic différentiel avec d'autres dermatoses neurophiliques, notamment le pyoderma gangrenosum et les dermatoses infectieuses, étaient facilement résolues par la confrontation de l'aspect histologique et des données clinicobiologiques. Les patients ont été traités par des anti-inflammatoires non stéroidiens en particulier par de l'indométacine à la dose de 150 mg/J la première semaine et 100 mg/J les deux semaines suivantes. Dans la majorité des cas la réponse était favorable avec rémission en quelques jours, dans les rares cas résistants ou répondant d'une manière incomplète on a eu recours au corticoïdes dans le cas du Sweet profond avec atteinte neurologique et à la colchicine dans un autre cas de Sweet typique avec atteinte articulaire. Trois patients n'ont pas reçu de traitement. Les patients étaient suivis sur des périodes variables allant de 3 mois à 4 ans. Le suivi reposait sur un interrogatoire, un examen physique et une numération formule sanguine. L’évolution était favorable chez 92% des patients avec résolution des signes généraux et locaux. Des récidives locales étaient rapportées dans 8% des cas. Quatre patients étaient perdus de vue. Parmi les patients qui n'ont pas reçu de traitement, l’évolution spontanée était favorable dans deux cas; le troisième était perdu de vue. Au cours de l’évolution, il n'y a pas eu de localisation neutrophilique extracutanée observée.

## Discussion

Le SS appartient au spectre des dermatoses neutrophiliques dont les plus fréquentes sont le pyoderma gangrenosum, la dermatose pustuleuse sous-cornée, l'erythema elevatum diutinum [3]. Le SS est la forme la plus fréquente des dermatoses neutrophiliques [4]. Il est défini par l'association de deux critères majeurs et de deux critères mineurs selon la classification de Su et Liu, modifiée par Von den Driesch [5]. Son incidence annuelle est évaluée à trois par million d'individus. Plus fréquent chez la femme, il prédomine à l’âge moyen de la vie mais peut survenir à tout âge [6]. Il survient rarement chez l'enfant (8%), sans prédominance de sexe [7]. La pathogénie du SS est multifactorielle [6]. L'association à des maladies infectieuses, des maladies auto-immunes, des néoplasies ou des médicaments plaide en faveur d'une réaction d'hypersensibilité [7]. Elle ferait intervenir des cytokines, l'interféron gamma et le TNF pour l'activation et le recrutement des neutrophiles [8]. Certains auteurs insistent sur le rôle de l'interleukine-1 (IL-1) et montrent l'efficacité du traitement par l'antagoniste du récepteur de l'IL-1 dans les cas réfractaires [9]. Cliniquement, le SS débute brutalement, parfois annoncé par des prodromes non spécifiques, respiratoires ou digestifs, avec une fièvre, des myalgies, des arthralgies, des douleurs abdominales ou une conjonctivite [4]. À la phase d’état, la fièvre est accompagnée d'une nette altération de l’état général. L’éruption est faite de plaques et de papules érythémateuses infiltrées, surélevées, bien limitées, à surface bosselée, de siège dermique et hypodermique. Ces plaques ne sont pas prurigineuses, mais sensibles et douloureuses. Il peut s'agir d'une lésion unique ou de lésions multiples, asymétriques. Elles prédominent au visage, au cou, aux membres supérieurs, mais peuvent atteindre tout le tégument [4, 6]. Les lésions ulcérées survenant au niveau des muqueuses sont souvent rapportées dans les formes associées à des hémopathies [7]. Ces lésions cutanées posent un problème de diagnostic différentiel notamment avec les maladies infectieuses, certaines vascularites ou maladies systémiques [7].

Les anomalies biologiques se traduisent par une élévation de la vitesse de sédimentation supérieure à 20 mm à la première heure, une élévation de la CRP, une hyperleucocytose à PNN supérieure à 70% [6]. Dans notre série, une hyperleucocytose avec polynucléose est notée dans 64%. Cependant, une anémie, une leucopénie et une thrombocytémie sont possibles, notamment dans les formes associées à des hémopathies [7]. La biopsie cutanée est nécessaire à la confirmation du diagnostic. Histologiquement, l’épiderme peut être normal, acanthosique ou être le siège d'une exocytose de PNN, parfois si intense qu'elle détermine la formation de pustules sous-cornées uniloculaires. Il peut exister un décollement sous-épidermique résultant d'un ‘dème massif [7]. Dans notre série, un œdème est noté dans 48% des cas. Au niveau du derme superficiel, il existe un oedème d'intensité variable. L'infiltrat se dispose en une large bande avec une tendance à la formation de nodules plus ou moins coalescents à renforcement périvasculaire [10]. Il peut s’étendre dans le derme profond, formant des amas plus ou moins denses de topographie périvasculaire et péri-annexielle. Cet infiltrat est majoritairement formé de PNN. Néanmoins, sa composition dépend du stade évolutif des lésions: un contingent de cellules lymphocytaires peut apparaître dans les stades débutants en particulier dans les formes associées à un syndrome myélodysplasique [11]. Actuellement, la présence d'une vascularite n'est plus un critère d’élimination du diagnostic [3]. En effet, une nécrose fibrinoïde de la paroi des vaisseaux et une leucocytoclasie peuvent être observées dans des formes typiques de SS. Dans une série de 37 cas de SS colligés par Jordaan, une vascularite leucocytoclasique et nécrosante est notée dans 18% des cas [12]. Malone et al, dans leur série de 21 cas, montrent que la vascularite n'est pas un processus immunitaire primitif, mais survient secondairement suite à la libération des métabolites toxiques libérés par des neutrophiles activés dans des lésions anciennes [13]. Il s'agit donc d'un signe témoignant de l'ancienneté des lésions. Dans notre série, une vascularite leucocytoclasique est notée dans 3 cas (12%). Au niveau de l'hypoderme, l'atteinte peut être septale, lobulaire ou mixte [6]. Ces formes hypodermiques des SS semblent plus fréquentes dans les SS associés à des hémopathies malignes, notamment aux syndromes myélodysplasiques. Le SS pose un problème de classement dans le spectre des dermatoses neutrophiliques qui constituent un continuum, avec des entités qui sont parfois associées [14] et s'accompagnent parfois de localisation extracutanées comme au cours du syndrome des abcès aseptiques [15]. Par ailleurs, une lésion infectieuse doit être éliminée telle que la cellulite, l’érysipèle, la tuberculose. Un prélèvement bactériologique peut s'avérer indispensable. Une fois le diagnostic posé, le problème essentiel sera la recherche, d'une part, de manifestations extracutanées, d'autre part, d'une maladie associée, essentiellement une hémopathie [3].

On peut distinguer plusieurs formes de SS. Les formes classiques ou idiopathiques sont les plus fréquentes (75%) [16]. Elles surviennent souvent chez la femme de la quarantaine et comportent presque toujours une polynucléose sanguine [3]. Le SS est paranéoplasique dans 10 à 20% des cas, accompagnant ou révélant une affection maligne qui est hématologique dans 85% des cas et une tumeur solide dans 15% des cas. La leucémie myéloïde aiguë est l'hémopathie la plus fréquemment diagnostiquée (85% des cas). Les tumeurs solides sont le plus souvent génito-urinaires, mammaires ou digestives [7]. Les SS para-inflammatoires sont associés à une maladie du groupe dysimmunitaire telle que la rectocolite ulcéro-hémorragique et la maladie de Crohn. Les infections les plus souvent rapportées sont celles des voies respiratoires supérieures. Les formes secondaires à une prise médicamenteuse incriminent plusieurs classes thérapeutiques et notamment les facteurs de croissance hématopoïétique [3]. Chez l'enfant et l'adolescent, la forme accompagnant une affection inflammatoire (33%) est la plus fréquente, suivie par la forme paranéoplasique (25%) [17]. Dans notre série, le SS était idiopathique dans 10 cas (40%) et parainflammatoire dans 10 autres cas (21,3%). Trois formes paranéoplasiques ont été notées et deux cas post médicamenteux. Le suivi était de durée variable entre 3 mois et 4 ans. Il s'est basé sur un interrogatoire, un examen physique et des examens complémentaires orientés selon les signes d'appel du patient. Au cours de l’évolution d'un SS, tous les organes et les muqueuses peuvent être le siège du même infiltrat neutrophilique aseptique et se manifester par des symptômes variés selon l'organe atteint. L'atteinte oculaire peut être la première manifestation du syndrome. Il s'agit d'une conjonctivite, d'une épisclérite ou une iridocyclite. L'atteinte articulaire est fréquente et très polymorphe. Elle survient dans 33 à 62% des cas. Il s'agit d'une arthrite aseptique qui peut être aiguë ou chronique, mono- ou polyarticulaire, atteignant les membres supérieurs ou inférieurs [7]. L'atteinte neurologique ou neuro-Sweet peut se manifester par une encéphalite ou une méningite et pose un problème diagnostic avec leneuro-Behçet. Les autres manifestations extracutanées sont rares et peuvent être pulmonaire, neurologique, cardiaque, osseuse [4]. L'approche thérapeutique dépend de l'extension et la profondeur des lésions, de l’état général du patient et des maladies associées. L’évolution spontanée peut être favorable en quelques semaines à quelques mois notamment dans les formes idiopathiques. Le traitement de la maladie associée, quand elle est curable, peut influencer favorablement le pronostic. La corticothérapie systémique à la dose de 0.5 à 1mg/kG/J avec décroissance lente et progressive en quelques semaines est le traitement de choix. La corticothérapie intralésionnelle et les dermocorticoïdes sont efficaces dans les formes localisées. D'autres alternatives thérapeutiques peuvent être proposées en première intention telles que la colchicine, indométhacine, iodure de potassium, dapsone, clofazimine, cyclines, ciclosporine, thalidomide, isotrétinoïne, anakinra, mais ces mollecules ont fait l'objet de publications que sur des cas isolés ou de très petites séries. Trois d'entre elles semblent toutefois particulièrement intéressantes: l'iodure de potassium (mais 2 cas de vascularites sévères ont été rapportées sous ce traitement), la colchicine à la dose de 1 à 1,5 mg/J et l'indométhacine à la dose de 150 mg/J la première semaine et 100 mg/J les deux semaines suivantes. Recommandés en première intention chez les personnes âgées, ou en cas de contre-indication aux corticoïdes bien que la rapidité d'action et le faible pourcentage de récidives observé avec les 2 dernières molécules sont des arguments pour une prescription plus large de première intention [18].

Les corticoïdes représentent là aussi le traitement de référence, à la dose de 0.5 à 1mg/kG/J. Ils sont très efficaces avec disparition rapide des symptomes. Les récidives sont cependant assez fréquentes à l'arrêt (>20%), et les formes chroniques ne sont pas rares (10% à 3 ans). Un certain nombre d'alternatives ont été proposées: colchicine, indométhacine, iodure de potassium, dapsone, clofazimine, cyclines, ciclosporine, thalidomide, isotrétinoïne, anakinra’ mais ces mollecules ont fait l'objet de publications que sur des cas isolés ou de très petites séries. Trois d'entre elles semblent toutefois particulièrement intéressantes: l'iodure de potassium (mais 2 cas de vascularites sévères ont été rapportées sous ce traitement), la colchicine à la dose de 1 à 1,5 mg/J et l'indométhacine à la dose de 150 mg/J la première semaine et 100 mg/J les deux semaines suivantes. Recommandés en première intention chez les personnes âgées, ou en cas de contre-indication aux corticoïdes bien que la rapidité d'action et le faible pourcentage de récidives observé avec les 2 dernières molécules sont des arguments pour une prescription plus large de première intention Dans notre expérience les AINS administrés en première intention avaient des résultats très satisfaisant incitant à préconisé cette option thérapeutique afin d’éviter une corticothérapie systémique étant une thérapie lourde avec beaucoup plus d'effets secondaires.

## Conclusion

Les résultats cliniques et histologiques des 25 patients atteints de SS dans notre étude sont généralement comparables à ceux publiés dans la littérature, avec quelques différences. Ce qu'il faut retenir c'est que Le syndrome de Sweet peut précéder ou révéler des maladies associées, ce qui nécessite une surveillance rigoureuse et prolongée.
